# What Brings Out the Best and Worst of People With a Strong Explicit Achievement Motive? The Role of (Lack of) Achievement Incentives for Performance in an Endurance Task

**DOI:** 10.3389/fpsyg.2020.00932

**Published:** 2020-05-26

**Authors:** Julia Schüler, Wanja Wolff

**Affiliations:** Department of Sport Science, University of Konstanz, Konstanz, Germany

**Keywords:** explicit achievement motive, motives, incentives, motor performance, underperformance

## Abstract

An explicit achievement motive is intuitively related to good performance. In contrast, the present paper directs attention to conditions where individuals with a strong explicit achievement motive display poor performance. We hypothesized that participants with a strong achievement motive perform worse in a bicycle ergometer task when task instructions lack achievement incentives than when the instructions include achievement incentives. Furthermore, we expected that, when achievement incentives are lacking, they show even worse performance than participants with a weak achievement motive. For the latter, we assumed that they are relatively unaffected by the achievement incentive content of the instructions. In a within-subject experimental design (*N* = 55) with two blocks (achievement incentives vs. lack of achievement incentives; each block consisted of three trials), our hypotheses were partly supported. The lack of achievement incentives brought out the worst (regarding performance), but the presence of achievement incentives did not bring out the best of participants with a strong achievement motive. In the discussion, we suggest how to improve future experimental achievement settings and reflect the results within the framework of the differentiation into implicit and explicit motives.

## Introduction

That a strong achievement motive, defined as “the capacity to derive satisfaction from the autonomous mastery of challenging tasks” (Schultheiss, 2008, p. 603), which in turn encourages people to seek out situations that allow measuring one’s standards of excellence (McClelland et al., 1953), is associated with high performance is not only intuitively plausible but also supported by research in sports (Elbe et al., 2005; Wegner et al., 2014; Gröpel et al., 2016) and in several other domains of life, for example, at the workplace (e.g., Andrews, 1967; McClelland and Franz, 1992), in learning contexts (e.g., McKeachie, 1961; O’Connor et al., 1966; Dahme et al., 1993; Schultheiss and Köllner, 2014), and in research laboratories with a broad variety of achievement tasks (to name but a few examples: Thurstone, 1937; Lowell, 1952; Biernat, 1989; Puca and Schmalt, 1999; Fodor and Carver, 2000; Brunstein and Hoyer, 2002; Brunstein and Maier, 2005; Pang, 2010).

In the present paper, we focus on *explicit* achievement motive, which has to be differentiated from *implicit* achievement motive due to differences in the behavior it influences, in the incentives that arouse the motives, and in motive development, to name a few examples. (For a detailed differentiation into implicit and explicit motives, see McClelland et al., 1989; see also discussion below.) The explicit achievement motive is a consciously represented part of one’s self-concept (“I am a person for whom it is important to perform well,” “It is important for me to demonstrate good performance”). According to previous theoretical considerations, the explicit achievement motive is highly relevant for performance in general (McClelland, 1980; Brunstein and Maier, 2005), as well as for performance in the sports and exercise context (Elbe and Wenhold, 2005; Elbe et al., 2005).

Motivation researchers share the view that the link between achievement motivation and performance stems from the interaction between the person’s achievement motive and characteristics of the situation (e.g., performance feedback), which is associated with the possibility to satisfy the achievement motive and, as a result, promises the experience of positive affects (for achievement: feelings of pride, being satisfied because one acts in accordance with one’s self-concept) (Brunstein and Heckhausen, 2008). In brief, the achievement motive is aroused by corresponding achievement incentives (Beckmann and Heckhausen, 2018a, b; McClelland, 1980).

The explicit achievement motive is incited in situations in which *social* incentives are present. Social incentives are characteristics of the situation such as demands and expectations that come from outside the task (e.g., from experimenters, teachers, coaches) rather than from the task itself (activity incentives) (for details about social and activity incentives, see McClelland et al., 1989). Further typical social achievement incentives, which we will also utilize in our study, are *verbal stimuli*. Bargh et al. (2001), for example, found that achievement-related words such as “win,” “master,” and “achieve” enhance performance. Also supporting this so-called “behavioral priming effect“(see also Breidebach and Gruber, 2019), Engeser et al. (2016) used excerpts from mathematics (Experiment 1) and language (Experiment 2) schoolbooks and found that semantic achievement primes enhanced performance in an arithmetic task and an anagram task, respectively. Following our line of argumentation that verbal stimuli leave *implicit* motives unaffected, the implicit achievement motive did not moderate the priming effect. In another series of studies, Engeser and Baumann (2014) found that verbally presented achievement primes indeed arouse explicit, but not implicit, achievement motivation (for further examples of achievement motive priming, see Breidebach, 2012, 2017).

A further example of verbal stimuli is achievement-related instructions in an experiment (McClelland et al., 1953; French, 1958). In the present study, we use task instructions for our experimental manipulation that stress the personal importance of performing well and demonstrating one’s competence.

The *presence* of achievement incentives is one “side” of the achievement motive × incentive interaction. But what about the other side of the interaction: How do people behave, when achievement incentives are *absent*? When social extrinsic incentives are missing, and when people do not feel responsible for the performance outcome (e.g., when performance depends on a device’s technical properties rather than on their performance; see calibration task below), then achievement behavior is not stimulated, and people with a strong achievement motive might perform even worse than people with weak achievement motives. The reason is that the explicit motives are built on the self-image of a person: “It operates as a cognitive regulator that shapes voluntary behavior in accordance with a person’s motivational self-view, mainly through its influence on cognitively based choices and explicit responses to social–extrinsic cues” (Brunstein and Maier, 2005, p. 206). One possible choice or explicit response is to put no effort in tasks that do not satisfy the achievement motive, for example, in a task without individual performance feedback, because this makes it impossible to assess and demonstrate one’s ability. In accordance with most motivation theories’ assumption, that goal characteristics and needs have a direct (McClelland et al., 1953; Wigfield and Eccles, 2000) or indirect (Brehm and Self, 1989; Wright, 2008) crucial role for investing energy in goal pursuit, people with a strong achievement motive are effective in effort regulation in the sense that they put the effort in tasks that are “worth” it (worth concerning motive-satisfying potential). When performance has consequences for one’s self-concept as an achievement-oriented and successful person, more effort should be mobilized to achieve high performance (Brunstein and Maier, 2005; Gendolla and Richter, 2010). They do not, however, waste time and effort in tasks that are “worthless” (= unable to satisfy the achievement motive). In sum, we assume that a strong achievement motive is associated with strategic effort investment. In contrast, for people with a weak achievement motive, achievement incentives are irrelevant, and their task performance might depend more strongly on other factors (e.g., compliance with the experimenter’s instructions).

In the present study, we experimentally designed instructions that either contain (achievement condition) or lack (calibration condition) achievement incentives to test the following interaction hypotheses concerning motor performance in a strenuous motor task: Participants with a strong achievement motive are expected to perform worse in an experimental condition that lacks achievement-related incentives than in a condition that includes achievement incentives. We furthermore assume that when a condition is void of achievement incentives, they perform even worse than participants with a weak achievement motive. When achievement incentives are present, they should perform better than the latter. We chose an endurance task (cycling on a bicycle ergometer) because physical performance can be accurately and continuously measured, and performance can be visualized to participants in real time. Further, as physical exertion creates a strong sense of effort, strategic effort investment (i.e., pacing) occurs even during short experimental tasks. We varied the difficulty of the endurance task (low, moderate, and hard levels of load) referring to an established power table (Coggans power table)^1^ and exploratorily examined whether load influences the hypothesized effect reported above.

## Materials and Methods

### Participants

Fifty-five students (31 women) from different faculties of a German university with a mean age of 23.67 years (*SD* = 5.09) participated in an experiment that was announced as a study that allegedly tested emotional well-being during an ergometer endurance task. We recruited participants using a university-internal platform, postings on blackboards, and advertisement in lectures. As exclusion criteria, we defined consumption of caffeine and tobacco less than 2 h before the experiment and alcohol or other drug intakes less than 12 h before the experiment. Further exclusion criteria were lower limb injuries and strenuous workouts in the last 12 h. Participants were informed that they will receive 10 Euros and that the study will last about 1 h.

### Procedure

The study design and material (e.g., information sheet, debriefing form) met the standards of the Ethics Committee of the authors’ university and were in line with the Declarations of Helsinki (World Medical Association, 2013). Participants were tested individually in a laboratory at the authors’ university. After being greeted by a female experimenter, participants read the information sheet and filled in the informed consent form. Then their height and body weight were assessed. While the experimenter prepared the bicycle ergometer (e.g., chose a load profile that fit participant characteristics, e.g., profile for men and women depending on body weight; see below), participants filled in a questionnaire containing the achievement motive measure (Unified Motive Scale, UMS, Schönbrodt and Gerstenberg, 2012) and a questionnaire that was used to test a hypothesis irrelevant for the present research (Self-Regulation Scale, Schwarzer et al., 1999)^2^.

After that, the experimenter adjusted the parameters on the bicycle according to the participants’ comfort (i.e., pedal and crank arm are parallel to the floor, while the knee and shank are perpendicular to the floor). Participants were then asked to start pedaling to familiarize themselves with the bike. They learned that they are expected to practice maintaining a given cadence that allegedly will be computed later from their individual scores (in fact, all cadences were 70 rpm) and tried it out during a familiarization phase. The cadence was displayed continuously on a screen in front of the participants while they were pedaling. The given cadence was clearly marked by a line so that participants could continuously observe deviations from the target cadence. It was explained that being precise – that is, keeping a specified cadence of 70 rpm – represents good performance. This should also include the reverse conclusion that poor performance is indicated by deviations from cadence (no matter whether higher or lower scores were achieved). A warm-up phase lasting 3 min followed. Participants were requested to inform the experimenter in case of any pain or discomfort during the course of the test and had the opportunity to ask questions about the whole procedure.

Then the actual test that consists of two blocks with three levels of load started. The first block consisted of half of the participants in the calibration condition and the other half in the achievement condition (random assignment to the order of condition). After a 5 min break, the groups received the respective other condition. The experimental conditions were implemented using written instructions for the cycling task. The achievement instructions contained positive evaluations of performance (which is a scoring category for the achievement motive in Winter’s scoring manual, Winter, 1994) (e.g., “optimal cadence,” “accuracy of your performance”) that unambiguously can be attributed to participants (it is about “YOUR individual performance”). As the incentive for people with a strong achievement motive is “to do something better” (McClelland, 1985), they need a tool to assess performance, for example, by receiving feedback on how well they are doing. In accordance with these theoretical considerations, previous research supported that people with a strong (implicit and explicit) achievement motive benefit more from feedback than participants with weak achievement motives (Fodor and Carver, 2000; Brunstein and Maier, 2005). We, therefore, announced an “individual performance log” in the instructions of the achievement condition. In contrast, in the calibration task, performance was framed as an accuracy assessment of the ergometer. With this, we intended to make participants feel responsible for their performance outcome in the achievement, but not in the calibration, condition (for a similar procedure, see McClelland et al., 1949). The concrete instructions for the achievement and calibration conditions are displayed in Table 1.

**TABLE 1:** Wording for the achievement and calibration conditions in the two separated blocks of the experiment.

	Block 1: Achievement		Block 2: Calibration
1.1	Let’s start with the test.	2.1	The ergometer needs a new calibration. Like this, we can be sure about the accuracy of the ergometer.
1.2	First of all, we will do three runs with different levels of difficulty	2.2	We are again performing three runs with different levels.
1.3	With these runs, we will measure the accuracy of YOUR task PERFORMANCE.	2.3	We will perform the same as before. But this time, we will test the ERGOMETER instead of you.
1.4	We can measure your performance accurately in the course and compare it to the rest of the participants.	2.4	This time, we are measuring the accuracy of our ergometer. We have to calibrate the ergometer to make sure it measures accurately.
1.5	Here you can see your personal performance line, which we have calculated for you during the pre-test (warming-up). The optimal cadence per minute for you is 70. This line here displays the 70 rpm cadence line. Your task is to keep the cadence of 70 rpm as constant as possible around (above or below) this line. Like this, we can exactly figure out your precision of riding a specific cadence. Go for it!	2.5	Here you can see a calibration profile presented by the software of the ergometer. The cadence is 70 rpm. This line here displays the 70 rpm cadence line. Your task is to keep the cadence of 70 rpm as constant as possible around (above or below) this line. Like this, we can figure out the accuracy of our ergometer. The calibration is starting soon!
1.6	*Afterward, you will receive your individual performance log. Here, you can see an example of an ergometer protocol. The performance log will look like this. It shows your performance over time and in comparison to the other participants.	2.6	*This time, this will be the test report for the ergometer. So, we will get a log with the deviation of the ergometer from its calibration line. Roughly the same will be our performance log for the ergometer. It shows the deviation of the ergometer compared to the calibration line.

The numbering is just for illustration of the experimental manipulation; participants read a running text. *A profile example was presented to show how the participant’s profile (/the bicycle ergometer’s profile) could look at the end. Participants were told that they would receive a printed version of their own profile (/the ergometer’s profile) at the end of the study.

The two ergometer blocks consisted of three levels of loads (low, moderate, hard) lasting 2 min each with a break of 1 min between each run. We used the Coggans power table^3^ to determine gender-specific power/weight (p/w) ratios for the low, moderate, and hard load levels. Female participants had to pedal at a p/w ratio of 1.6 for the easy, 2.1 for the moderate, and 2.6 for the hard load levels, and male participants pedaled at 2.4, 3, and 3.6 p/w for the easy, moderate, and hard levels. The loads were calibrated in such a way that even untrained participants would be able to complete 2 min of the hard load. After the cycling part, the participants answered two questions checking whether they were aware of the hypotheses (What do you think was the real aim of the study? How do you think the two sessions were connected?). No participant realized the study’s intention. Then they stated whether they consumed any drugs, caffeine, or alcohol prior to the study (exclusion criteria). They then received a debriefing form and their payment and were thanked and dismissed.

### Measures

We used a German version of the Unified Motive Scale (UMS-10, 54 items, Schönbrodt and Gerstenberg, 2012) that assesses a broader range of self-attributed motives. Reports about the UMS reliability and validity can be found in Schönbrodt and Gerstenberg(2012); see also Sariyska et al. (2019). The **achievement motive** scale, which is relevant for our research question, consists of two statements (i.e., “I am appealed by situations allowing me to test my abilities, “My goal is to do at least a little bit more than anyone else has done before”), which require an agreement rating on a six-point rating scale ranging from 1, strongly disagree, to 6, strongly agree, and eight goals (e.g., “continuously improve myself,” “personally producing work of high quality,” “opportunities to take on more difficult and challenging goals and responsibilities,” “personally doing things better than they have been done before,” “opportunities to create new things,” “projects that challenge me to the limits of my ability,” “continuously new, exciting, and challenging goals and projects,” “maintaining high standards for the quality of my work”), which require an importance rating (1, not important to me, to 6, extremely important to me).

We used a Cyclus2 ergometer (by RBM Elektronik-automation GmbH, Leipzig, Germany) to assess **motor performance**. Cycling data were measured at a sampling rate of 2 Hz and with a set true gear ratio of 53/14. Participants were asked to keep the cadence as close as possible to around 70 rpm. In asking participants to keep this pre-defined cadence, we used accuracy rather than a “the more the better” (e.g., higher cadence indicates better performance) criterion of performance. We therewith ensured that the participants could not overspend themselves at the beginning (and have to abort their participation in the rest of the experiment due to physical exhaustion). In addition to these laboratory experimental reasons, motor precision is important to be analyzed, because it is crucial for an accurate execution of movement, which is important for injury prevention (for a similar reasoning, see Hirsch et al., 2020, March 17). A further reason is that motor response precision requires high exertion of self-control. Manohar et al.(2015, p. 1707) called this fact “cost of control,” which means that exerting control to improve response precision itself comes at a cost (the costs to attenuate a proportion of intrinsic neural noise) (Giboin et al., 2019). Furthermore, keeping a specific cadence comes closer to Brunstein and Maier (2005) findings that the explicit achievement motive predicts the continuation of a task (keep on going) rather than the performance itself (e.g., peak performance).

Our choice of the level of cadence that we asked participants to keep (70 rpm) was based on studies that analyzed what cadence non-cyclists prefer when they are free to choose (Whitty et al., 2009). Non-cyclists freely chose levels of the cadence of about 80 rpm that they report to be comfortable, which, however, were significantly higher than the most economical cadence (in terms of metabolic economy, 50 rpm). Cycling at a cadence above or below the most economical cadence of 50 rpm was experienced as strenuous, indicated by higher ratings of perceived effort (RPEs). We chose a cadence (70 rpm) that deviates from the expected preferred cadence of 80 rpm, to ensure that participants were not likely to automatically gravitate to our target cadence.

In our experiment, deviations from a cadence of 70 rpm were squared and averaged for each load and condition. Thus, higher scores in “Cadence” represent poorer performance.

## Results

### Description of Data and Their Relationships

Table 2 depicts means and standard deviations for participants’ motor performance (Cadence) for the achievement goal and calibration conditions, each for an overall score and for low, moderate, and hard loads. The achievement motive (*M* = 3.48, *SD* = 0.723) is positively correlated with deviation from cadence in the achievement condition (*r* = 0.31, *p* = 0.021) as well as in the calibration condition (*r* = 0.38, *p* = 0.004). Cadence scores of both conditions were highly related with *r* = 0.37, *p* < 0.005.

**TABLE 2:** Means and standard deviations (in brackets) for Cadence for the Achievement and Calibration Conditions separated for different levels of load.

	Achievement condition				Calibration condition			
Load	Overall	Low	Moderate	Hard	Overall	Low	Moderate	Hard
Cadence	2.27	2.24	2.13	2.45	4.07	2.97	4.14	5.09
	(4.21)	(3.95)	(4.37)	(4.32)	(8.82)	(5.66)	(9.1)	(11.7)

*Cadence means squared deviations from a cadence of 70 rpm, and thus, higher scores mean poorer performance.*

### Testing the Hypotheses

To test the hypothesis that participants with a strong achievement motive perform worse in the calibration condition than in the achievement condition (goal conditions were nested within participants), we conducted a hierarchical multi-level regression analysis using the statistical software R (R Core Team, 2017) and the lme4 package (Bates et al., 2015). We chose a hierarchical multi-level model to better account for the fact that people differ but that these differences do not stem from the experimental manipulation. The regression model was built by sequentially adding predictors after an intercept-only model, and its random intercept had been specified (*baseline model*). To create the *main effect model*, we added the single predictors (Condition, ACHmotive) to the baseline model with Condition allowed to vary between participants. The *interaction model* consists of the single predictors and the two-way interaction (Condition × ACHmotive). This procedure allowed us to compare all stages of model specification and figure out whether the interplay between ACHmotive and Condition can explain variance in addition to the single variables.

**FIGURE 1:**
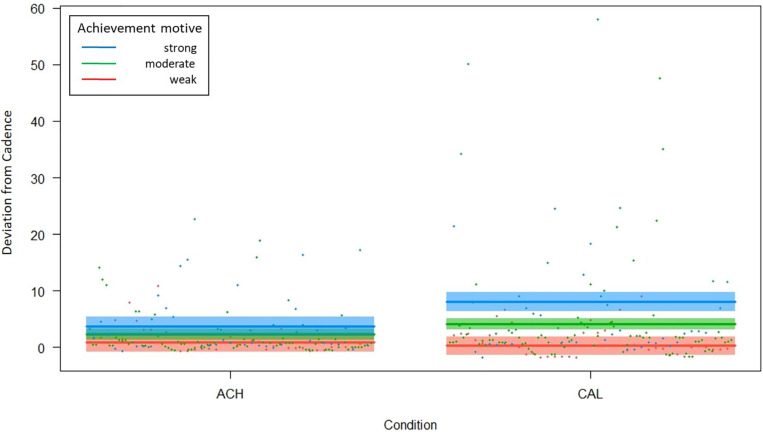
Illustration of the Condition × Achievement Motive interaction. Note. Cadence means squared deviations from a cadence of 70 rpm, and thus, higher scores mean poorer performance. ACH: Achievement condiction, CAL: calibration conditions.

The interaction model summary showed that the ACHmotive significantly [*b* = 1.538, *t*(53) = 2.371, *p* = 0.021, *d* = 0.320] and the interaction marginally [*b* = 2.755, *t*(53) = 1.973, *p* = 0.054, *d* = 0.266] predicted performance. Condition was not a significant predictor [*b* = −7.800, *t*(53) = −1.571, *p* = 0.122, *d* = 0.212]. An ANOVA testing the *main effect model* against the *interaction model* revealed a significant effect [χ^2^(1) = 3.898, *p* = 0.048] indicating that the interaction model significantly improved the fit over the main effect model. Figure 1 illustrates the nature of the interaction.

In accordance with the hypothesis, participants with a strong achievement motive (blue lines and dots) performed worse in the calibration than in the achievement condition. Also as expected, in the calibration condition, they performed worse than participants with a weak motive (red lines and dots). Unexpectedly, however, participants with strong and weak achievement motives did not significantly differ in the achievement condition.

### Exploratory Analyses

To examine whether the load of the task (easy, moderate, high) could improve the fit of the model, we examined a *main effect model* (Condition, ACHmotive, Load), a *two-way interactions model* (main effect model plus the three two-way interactions), and a *three-way interaction model* (two-way interaction model plus Condition × Load × ACHmotive interaction). Neither the three-way interaction nor the two-way interactions were significant^4^.

## Discussion

We aim to make use of the primacy effect (Ebbinghaus, 1913; Murdock, 1962) and start our discussion with the results that support our hypotheses. A hierarchical linear regression analysis revealed that considering the interaction between incentive content of task instruction and participants’ achievement motive contributes to explain performance. The interaction itself was only marginal, but the interaction model contributes significantly when compared to the main effect model. To sum, although we assumed this interaction effect to be stronger, it is a least partly in conformity with the fundamental hypothesis in motive research that motives interact with characteristics of the situation to predict behavior (Brunstein and Heckhausen, 2008). However, the pattern of the interaction only partly supported our assumptions. In line with the hypotheses, participants with a strong achievement motive performed worse in an experimental condition that lacks achievement incentives (calibration condition) than in a condition that includes achievement incentives (achievement condition). Also as expected, in the calibration condition, they performed worse than participants with weak achievement motives. This supports our assumption that people with strong achievement motives perform badly when the effort that has to be invested in a task is not worth the effort because it does not promise motive satisfaction. We speculate that this “lazy” behavior is a clever strategy to invest one’s effort efficiently, for example, by not “wasting” it on an unrewarding task (but saving energy for highly rewarding tasks).

Also in accordance with our hypotheses, people with a weak achievement motive, who are not dispositionally interested in demonstrating performance, showed relatively robust performance across the two experimental conditions. Their performance behavior was fully in line with the instructions given by the experimenter. It seems that agreeableness and compliance with experimental instructions had guided their behavior. This is in contrast to participants with a strong achievement motive who appear to have regulated their effort depending on the incentive content of the instructions. Unexpectedly, the assumption made for the achievement condition was not supported by the data. Here, participants with a strong achievement motive did not perform better than participants with a weak achievement motive. An obvious reason is that participants with a weak achievement motive performed extremely well (very small deviations from cadence), and surpassing this level is virtually impossible. However, people with strong achievement motives should at least show the same performance. One explanation why they don’t might be that – although we derived the components of the task instructions theoretically – we failed to create an experimental setting that incites the explicit achievement motive well. It might be that announcing an individual performance log did not directly promise to fulfill the desire to demonstrate one’s performance. We should have extended the announcement by adding the information that their ergometer protocol would be published and therefore would be visible for others. Furthermore, as has been shown in the literature of achievement goal priming, using words as primes is not always effective (Harris et al., 2013). In addition, the effects of self-set goals might outweigh those of the priming, or stronger effects would be revealed from field rather than from laboratory settings (Chen et al., 2020).

It could also be that we did not only fail to incite the achievement motive in the achievement condition but that we even demotivated people with a strong achievement motive (as the positive correlation between achievement motive and deviation from cadence would support) by accidentally restricting their autonomy. Although the following considerations about autonomy and achievement motive refer to the implicit achievement motive, they could also apply to the explicit achievement motive: The incentive for people with a strong achievement motive is the autonomous mastery of challenging tasks (McClelland et al., 1953). As pointed out by Schultheiss(2008, p. 603), however, if people with a strong achievement motive “cannot chose and solve such tasks on their own terms, but are given explicit advice and direction on how to do it, they are likely to leave the field and invest no effort in the task” (see also Spangler, 1992). Reconsidering the instructions in the achievement condition, we might have put too much pressure on them by clearly directing them (“Your task is to keep the cadence of 70 rpm”) and by demonstrating control by announcing that the experimenter monitors compliance with the instruction (“We can exactly figure out your precision of riding a specific cadence”). Future studies should give people with a strong explicit achievement motive more scope to choose the challenging task themselves. Most importantly, speculations about if and how the experimental manipulation had worked can be simply avoided by manipulations checks (e.g., asking participants how motivated they felt in each condition).

Furthermore, physical performance is only one single aspect of one’s capability, and some (rather untrained) participants with strong achievement motives might not feel incited by this domain-specific performance setting. To avoid possibly domain-specific arousal of the achievement motive, we suggest adding the information to future study instructions that showing good performance in this endurance task is known to be an indicator of good performance in other domains of life (academia, workplace).

### Limitations and Further Research Tasks

Our research leaves further open research questions that we aim to address in the following. In our study, we assessed explicit achievement motives because they are known to be triggered by social achievement incentives such as expectations from the experimenter, coaches, sport education teachers, and fitness instructors. However, next to the explicit achievement motive, the implicit achievement motive (unconsciously represented, based on affects), which is characterized by the enjoyment of challenging tasks and the anticipation of feeling proud after success (McClelland et al., 1953; Brunstein and Heckhausen, 2008), also plays a crucial role in sport performance (Gröpel et al., 2016). Implicit and explicit motives differ, among other things, in the incentives (social vs. activity incentives) that trigger them and lead to motive-relevant behavior. On the theoretical level, there is a clear-cut differentiation in regard to what type of incentives arouse which type of motive. Experimentally (and in everyday life), it is challenging to create incentives that map perfectly on this differentiation. Social and activity incentives cannot easily be isolated from each other. In the present laboratory study, it is highly probable that not only the result of the motor task motivated people to perform well or to save energy (explicit incentive) but also characteristics of the task itself. People with a strong *implicit* achievement motive might have been also attracted by, for example, the possibility to exceed their physical comfort zones and master a physically challenging task, regardless of the outcome of the activity (and therefore also regardless of being in the achievement or control group). We did not assess the implicit achievement motive and therewith cannot control for its influence on performance. A second way in which the implicit achievement motive might have undermined the hypothesized results is by being incongruent with the explicit achievement motive. The disconcordance between implicit and explicit motives causes impairment of well-being and motivation (Brunstein et al., 1998; Brunstein, 2010; Schüler, 2010) and requires self-control (Kehr, 2004; Schüler et al., 2019). However, all these are crucial for high performance in a strength endurance task.

Schüler (2010) took an even more extended perspective and examined the interplay between implicit and explicit achievement motives as well as achievement incentives. She found that achievement motive incongruence exerted stronger negative effects when individuals act in situations in which achievement incentives are present and arouse the conflict between the two motives. For the present study, this applies to the achievement condition but not the calibration condition. In brief, it might be that motive incongruence undermined the expected results only in the achievement condition (in which we found unexpected results) and left performance in the control condition (in which we found expected results) unaffected. Schüler (2010), however, analyzed effects on flow experience, and it is pure speculation (although theoretically plausible) that her findings also apply to motor performance. If we briefly accept this speculation as true, it still has to be explained why in the achievement condition, the motive incongruence reduced performance of people with a strong explicit achievement motive (and a weak implicit motive, Type 1 of motive incongruence) but not performance of people with a weak explicit achievement motive (and a strong implicit achievement motive, Type 2 of motive incongruence), who maintained their performance (see Figure 1). This can only be answered with another speculation. If a strong explicit achievement motive is not supported by the energy that is exerted in connection with a high implicit motive (Type 1 of incongruence), but this “energizing function” of implicit motives (McClelland et al., 1953) is needed for a strenuous endurance task, performance should be reduced. Type 2, in contrast, is characterized by a high implicit motive that remains unsatisfied, because a corresponding high explicit motive is lacking that elicits achievement-relevant behavior. In our laboratory session, however, the “achievement behavior” (holding a specific cadence) is pre-defined by the experimenter rather than initiated by the explicit achievement motive. The explicit achievement motive might not come into play here, and thus, the negative effects of this type of incongruence might therefore not be evinced. This explanation, however, is built on two speculations and has to be tested empirically.

Also, in reality, sport and exercise settings probably require a mixture of “respondent behavior” (McClelland, 1980) triggered by explicit motives and “operant behavior” triggered by implicit motives so that high standards of excellence can be achieved. Performance in a marathon race, for example, depends on the choice to participate in a large city marathon, for example, to fulfill the expectations of the running group and to demonstrate one’s high-performance capacity to the public (social incentives, respondent behavior), and it depends on activity-inherent incentives, such as liking to run and enjoying that one’s performance improves over time, that ensure long-term adherence to one’s training program (activity incentives, operant behavior). Therefore, one imperative for future research is to assess behavior in settings that are closer to sports reality and allow, for example, spontaneously generated behavior (operant behavior) and examine its interplay with respondent behavior. Furthermore, more complex incentive patterns (e.g., social and activity incentives) and the assessment and arousal of implicit and explicit achievement motives have to be considered to overcome the artificiality of the laboratory and to better understand the complex interplay of implicit and explicit motives.

A further task for future research is to assess the mechanism that we assume to underlie the underperformance of people with strong achievement motives. The “worth” of performance behavior outcome can be assessed by asking participants how much they value the calibration task and the achievement task, respectively. Additionally, the willingness to invest effort has to be assessed with objective measures (cardiovascular effort indicators) or by assessing the self-reported willingness to invest effort.

## Conclusion

To make use of the recency effect (Ebbinghaus, 1913; Murdock, 1962), we would like to close with a positive statement. Experimenters, coaches, sport education teachers, and fitness instructors can easily assist people by paying careful attention to the instructions they give. Although our study has not figured out what brings out the best from people with a strong achievement motive, it shows what instructions should be avoided because they harm people with a strong achievement motive. Practitioners in the sport context, for example, are recommended to avoid task instructions that do not contain any achievement incentives. They might function for some people who might exercise only to meet the expectations of others (in our study, people with a weak achievement motive who complied with instructions of the experimenter) but would discriminate against people with a strong achievement motive. But also for the former, the longer-term perspective does not look good: According to Self-Determination Theory (Deci and Ryan, 1985), external regulation (perform a behavior because somebody else wants me to do it) and introjected regulation (do it because you feel obligated) are less good predictors for the maintenance of sport and exercise over a longer time (for an overview, see Hagger and Chatzisarantis, 2008). We encourage researchers and people in the applied setting to consider individual differences in motives. This might help to better understand phenomena such as the assumed unwillingness to invest effort and the underperformance of people who are expected to perform well.

## Data Availability Statement

The raw data supporting the conclusions of this article will be made available by the authors, without undue reservation, to any qualified researcher.

## Ethics Statement

The studies involving human participants were reviewed and approved by Ethical committee of University of Konstanz. The patients/participants provided their written informed consent to participate in this study.

## Author Contributions

JS and WW conceived the study design, conducted the study, and performed the statistical analyses together. JS wrote the first draft of the manuscript. WW revised and finalized the manuscript.

## Conflict of Interest

The authors declare that the research was conducted in the absence of any commercial or financial relationships that could be construed as a potential conflict of interest.

## Footnotes

1 https://www.trainingpeaks.com/blog/power-profiling/

2 Further variables were assessed that are not relevant for the present research question (weight, height, perceived exertion).

3 https://www.trainingpeaks.com/blog/power-profiling/

4 We conducted a power analysis (using GPower 3.1; Faul et al., 2009) for a design with lower statistical power (no multi-level design). This analysis for an ANOVA with repeated measures testing a 2 (condition vs. achievement) ^∗^ 3 (easy vs. moderate vs. high) design (effect size = 0.25, alpha = 0.05, power = 0.80) revealed an ideal sample size of 44 participants (actual power: 0.9557).
